# Influence of maximum diameter on fine-needle aspiration biopsy outcomes in ACR TI-RADS 5 thyroid nodules

**DOI:** 10.3389/fendo.2024.1374888

**Published:** 2024-05-14

**Authors:** Shi-Liang Cao, Wan-Ying Shi, Yi-Ru Niu, Zhen-Long Zhao, Ying Wei, Jie Wu, Li-Li Peng, Yan Li, Ming-An Yu

**Affiliations:** ^1^ Department of Interventional Medicine, China-Japan Friendship Hospital, Beijing, China; ^2^ Department of Epidemiology and Health Statistics, School of Public Health, Capital Medical University, and Beijing Municipal Key Laboratory of Clinical Epidemiology, Beijing, China; ^3^ Pathology Department, China-Japan Friendship Hospital, Beijing, China

**Keywords:** fine needle aspiration, thyroid nodule, restricted cubic spline (RCS), diagnosis, ultrasound

## Abstract

**Introduction:**

Fine needle aspiration (FNA) biopsy is a widely accepted method for diagnosing thyroid nodules. However, the influence of maximum diameter (MD) of ACR TIRADS 5 (TR5) thyroid nodules on the FNA outcomes remains debated. This study examined the influence of MD on the FNA outcomes and investigated the optimal MD threshold for FNA in TR5 nodules.

**Methods:**

We conducted a retrospective analysis of 280 TR5 thyroid nodules from 226 patients who underwent FNA from January to June 2022 in our department. Probably malignant (PM) group was defined as Bethesda V in cytopathology with confirmed BRAF V600E mutation or Bethesda VI, the other cytopathology outcomes were defined as probably benign (PB) group. We examined factors influencing malignant cytopathology outcomes and determined the optimal MD threshold for FNA in TR5 nodules using logistic regression and restricted cubic spline (RCS) analysis.

**Results:**

Among these nodules, 58.2% (163/280) had PM outcomes. The PM group had a significantly larger MD than the PB group [6.5mm (range 5.0-8.4) vs. 5.3mm (range 4.0-7.0), p < 0.001]. In multivariate logistic regression fully adjusted for confounders, MD was significantly associated with PM outcomes [odds ratio 1.16, 95%CI 1.05-1.31; p = 0.042]. The highest quartile of MD had a greater likelihood of PM outcomes compared to the lowest quartile [odds ratio 4.71, 95% CI 1.97-11.69, p = 0.001]. The RCS analysis identified 6.2 mm as the optimal MD threshold for FNA in TR5 nodules.

**Conclusion:**

MD significantly affects the probability of malignant outcomes in FNA of TR5 thyroid nodules. A MD threshold of ≥6.2mm is suggested for FNA in these nodules.

## 1 Introduction

Thyroid nodules is a worldwide prevalent diseases with the incidence ranging from 5 to 68% in the ultrasound examination or palpation depending on iodine supply, the age of the population studied (1–5). Moreover, subsequent examinations or treatments reveal that 5-15% of these nodules are malignant (6, 7). Consequently, the prompt differentiation of malignant thyroid nodules from benign ones is of paramount importance.

Fine needle aspiration (FNA) biopsy is recognized as a minimally invasive and highly accurate technique for the pathological diagnosis of thyroid nodules. This method is endorsed by almost guidelines concerning the management and treatment of thyroid nodules (2, 8, 9). Various factors, including age, sex, calcification, echogenicity, composition, as well as the maximum diameter (MD) and depth of the nodules, could influence the outcomes of FNA (10–15). Among these, the MD is an significant and important influencing factor (10, 15–18). Nevertheless, there is still absence of the universally recognized optimal MD threshold for FNA in suspicious thyroid nodules (9), which leads to variability in clinical management decisions. The American Thyroid Association recommended the FNA for high or intermediate suspicion thyroid nodules that are 1 cm or larger (2), aligning with recommendations from other guidelines (8, 9).

It merits emphasis that papillary thyroid microcarcinoma (PTMC), characterized as papillary thyroid carcinoma with a size under 1 cm, maintains a degree of invasiveness and a risk of lymph node metastasis. Prior research indicates that the incidence of lymph node metastasis in PTMC ranges between 13.5-37.0% (19–21). Thermal ablation emerges as an efficacious and minimally invasive option for treating thyroid nodules, and is recommended for the treatment of selected PTMC cases confirmed by preoperative FNA in various guidelines (22, 23). Given this context, it becomes crucial to comprehensively assess the efficacy of FNA in evaluating thyroid nodules smaller than 1 cm. Additionally, determining the optimal MD threshold for administering FNA on these suspicious nodules warrants thorough exploration.

In the present study, clinical and imaging information of ACR TIRADS 5 (TR5) thyroid nodules underwent FNA was summarized, the influence factors of FNA were evaluated. Subsequently, the study delves into the relationship between the MD of these nodules and the results of FNA, aiming to identify the potential optimal MD threshold for FNA.

## 2 Methods

### 2.1 General information and patient enrollment

This retrospective, single-center study was approved by the ethics committee of China-Japan Friendship Hospital. Written informed consent was obtained from all participants. Written informed consent for the publication of data was waived because the examination results and radiological data of patients were published anonymously. Data of patients who underwent FNA for thyroid nodules between January 2022 and June 2022 were summarized.

**Figure 1:**
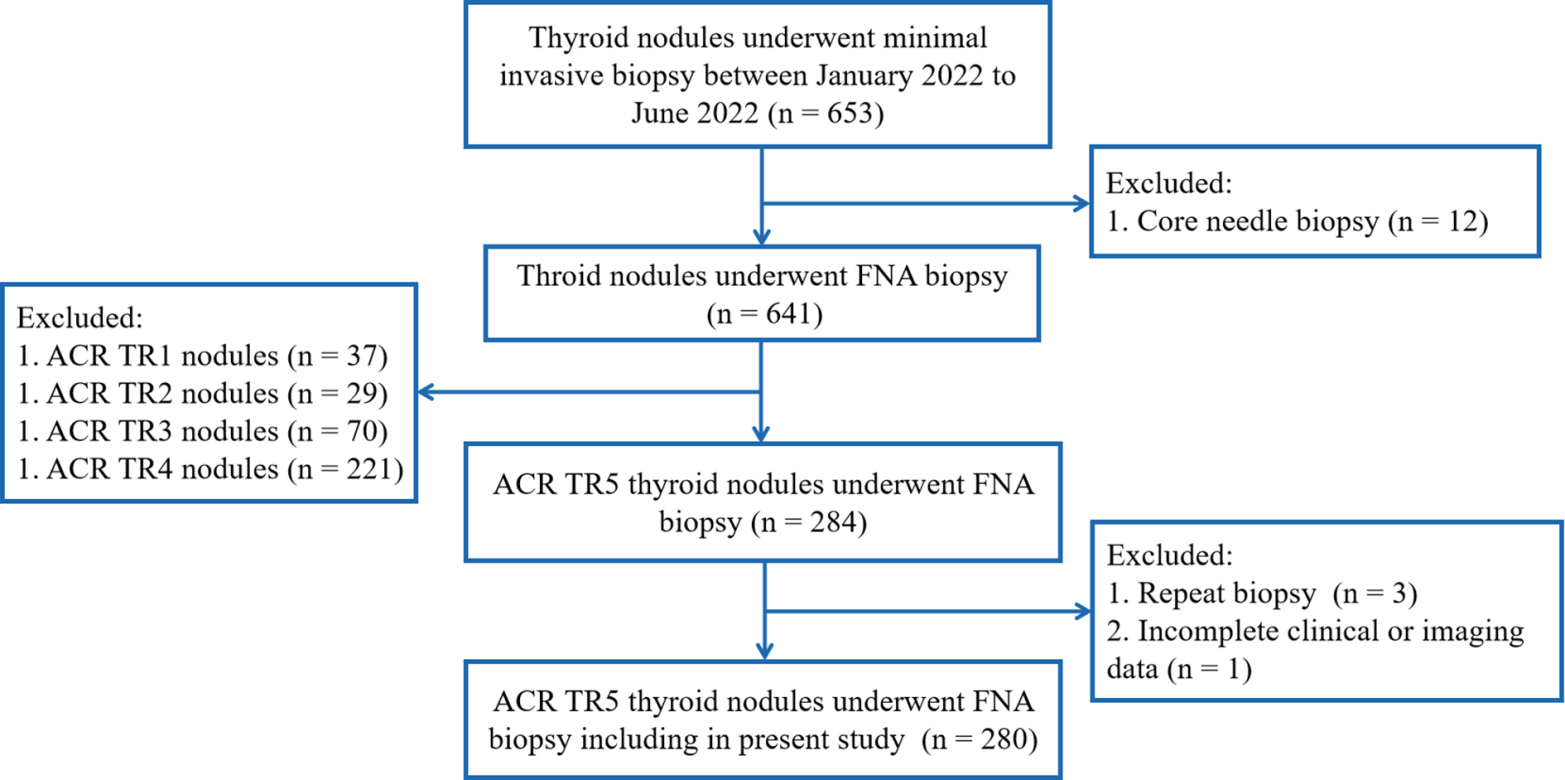
Flow chart and patients’ inclusion and exclusion criteria.

The inclusion criteria included: (1) the thyroid nodules underwent FNA biopsy, and (2) ACR TR5 thyroid nodules in ultrasound evaluation. Exclusion criteria were as follows: (1) repeat FNA on same thyroid nodules; (2) core needle biopsy of thyroid nodules, and (3) incomplete clinical or imaging data (**Figure 1**).

### 2.2 Preoperative evaluation and ultrasound-guided FNA

The ACR TIRADS was utilized to evaluate and classify the thyroid nodules in present study (9). Two doctors, each with over three years of experience in thyroid nodule ultrasound examination, conducted the evaluations. In cases of differing opinions, a senior doctor provided the final assessment. The ultrasound-guided FNA of thyroid nodules was performed by a doctor with a minimum of three years of clinical experience.

**Figure 2:**
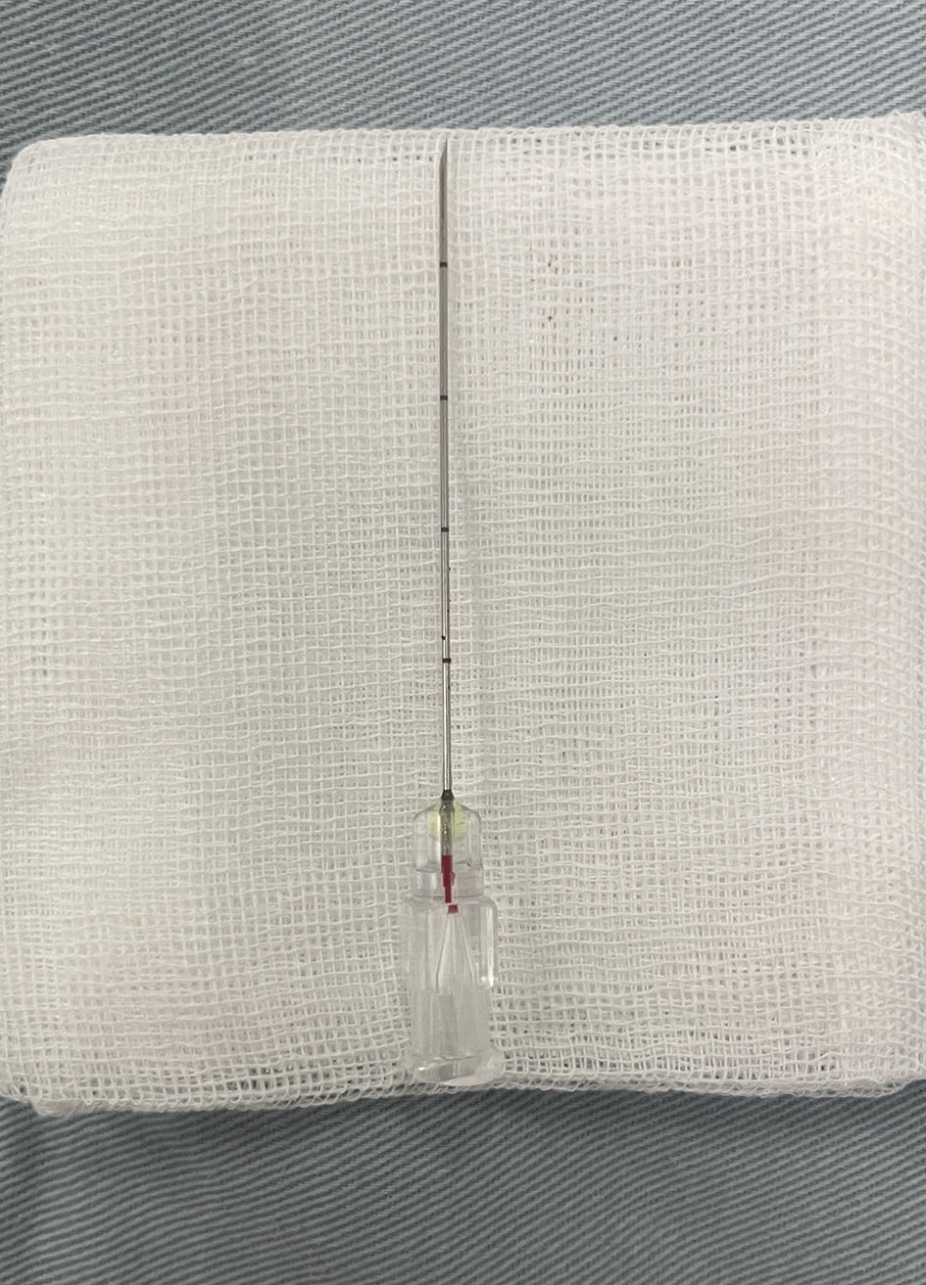
A trace of blood in transparent hub of 23G fine needle.

Prior to FNA, patients underwent laboratory tests including routine blood test, coagulation function tests, and calcitonin measurement. The preoperative evaluation and FNA were conducted using either the LOGIQ E9 scanner (GE Healthcare, Milwaukee, USA) or the Canon Aplio i900 (Canon Medical Systems, Otawara, Japan), both equipped with high-frequency linear array probes (9-18 MHz). Patients were positioned supine, and the anterior neck region was sterilized. Following local anesthesia, a 23G fine needle (Biopsy needles, Gallini S.R.L, Via S. Faustino, Modena, Italy) was precisely inserted into the center of the targeted thyroid nodules under ultrasound guidance. The needle core was withdrawn, and the needle sheath was then inserted into and retracted from the nodules several times without suction until the transparent needle hub showed a trace of blood (**Figure 2**) or the insertion was repeated more than 10 times. Post-procedure, the puncture site was compressed manually for 30 minutes to prevent hematoma formation.

Each targeted nodule was sampled thrice for FNA, followed by smear examination and liquid-based cytological testing. The eluate from the fine needles was analyzed for mutations in the B-type Raf kinase (BRAF) V600E gene.

### 2.3 Pathological outcomes

Pathological examinations and reporting were conducted by a senior pathologist specializing in thyroid cytopathology with three years of experience. The 2017 Bethesda System for Reporting Thyroid Cytopathology (TBSRTC) guided the pathological reports for thyroid FNA specimens (6), which include: (I) non diagnostic or unsatisfactory; (II) benign; (III) atypia of undetermined significance or follicular lesion of undetermined significance; (IV) follicular neoplasm or suspicious for a follicular neoplasm; (V) suspicious for malignancy; (VI) malignant.

In our study, the thyroid nodules with Bethesda V in cytopathology and confirmed mutation of BRAF V600E were regarded as malignant nodules. These nodules, along with the nodules of Bethesda VI, were defined as malignant cytopathology outcomes and grouped into probably malignant (PM) group. Other thyroid nodules were grouped into probably benign (PB) group.

### 2.4 Study variables

**Figure 3:**
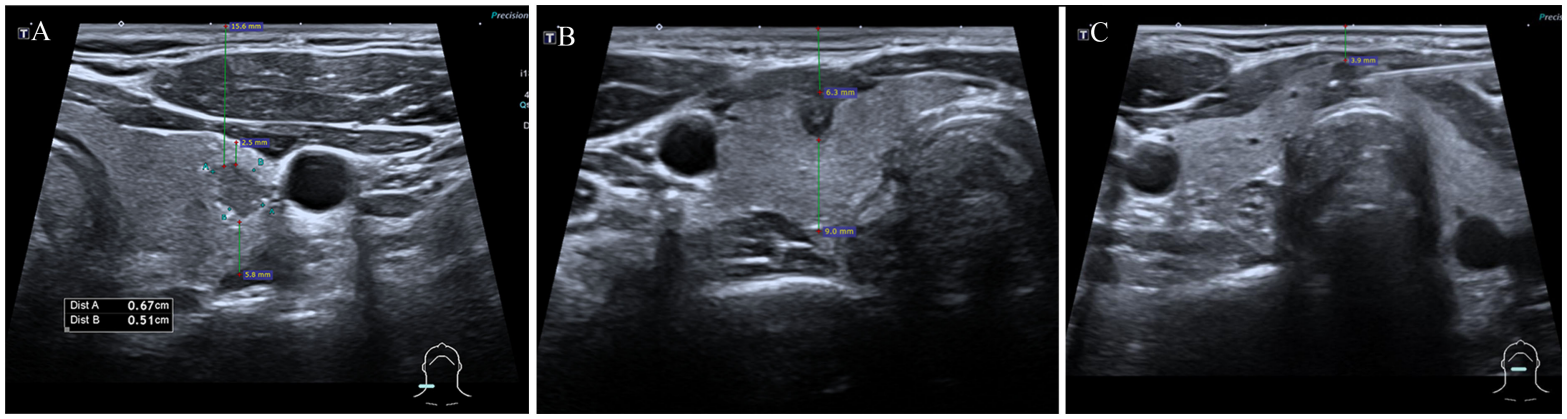
Measurements of thyroid nodules’ depth, anterior, and posterior distances. The depth of nodules refers to the vertical distance of nodules from the skin surface, anterior distance refers to the distance of nodules from the thyroid’s anterior capsule, posterior distance refers to the distance from the thyroid’s posterior capsuled. **(A)** depth: 15.6mm, anterior distance: 2.5mm, posterior distance: 5.8mm; **(B)** depth: 6.3mm, anterior distance: 0mm, posterior distance: 9.0mm; **(C)** depth: 3.9mm, anterior distance: 0mm, posterior distance: 0mm.

Demographic and clinical data of patients undergoing FNA were documented, including sex, age, and the number of nodules. Ultrasound images of the targeted thyroid nodules were reviewed, and characteristics such as composition, echogenicity, shape, margin, and echogenic foci were noted. Measurements including the MD, the vertical distance of nodules from the skin surface (depth), the distance of nodules from the anterior capsule of the thyroid (anterior distance), and the distance from the posterior capsule of the thyroid (posterior distance) (**Figure 3**) were taken and recorded. The presence of Hashimoto’s thyroiditis background in ultrasound imaging was noted due to its potential influence on nodule observation, characterized by heterogeneous echogenicity in the thyroid. Additionally, the dominance of the hand used for FNA was recorded. All variables and definitions used in the study are presented in **Table 1**.

**Table 1:** The variables included in model and definition.

Variables	Definition
Sex	Female Male
Age	Age(Year)
Number of FNA	Number of nodules underwent FNA simultaneously in a patient
Composition	Mixed: mixed cystic and solid Solid: solid or almost completely solid
Echogenicity	Isoechoic Hypoechoic and very hypoechoic
Shape	Taller than wide Wider than tall
Margin	Smooth Ill-defined Irregular: including lobulated Extension: extra-thyroidal extension
Echogenic foci	None: none or large comet-tail artifacts Macrocalcification Peripheral calcification Punctate echogenic foci
Hashimoto thyroiditis	Yes: There was Hashimoto’s thyroiditis background in ultrasound imaging. No
Maximum diameter (mm)	The max diameter of targeted nodules in ultrasound images.
Depth (mm)	The distance of targeted nodules from skin surface.
Anterior distance (mm)	The distance of targeted nodules from anterior capsule of thyroid.
Posterior distance (mm)	The distance of targeted nodules from posterior capsule of thyroid.
Position	Left lobe Right lobe Isthmus
Hand used for FNA	Dominant hand Non dominant hand

### 2.5 Statistical analysis

Continuous variables are presented as means and standard deviations (mean ± SD) for normally distributed data and as medians and interquartile ranges (IQR) for non-normally distributed data. Discrete variables are shown as frequencies and percentages. The Chi-square test was applied to compare discrete variables. For continuous variables, the Student’s t-test was used for normally distributed data, while the Mann-Whitney U test was employed for non-normally distributed data. Differences in baseline variables were analyzed using the CBCgrps package in R (24).

The present study explored the relationship between the MD of thyroid nodules and malignant outcomes in FNA using binary logistic regression. Model 1 did not adjust for any covariates; Model 2 was adjusted for age, gender, and ultrasound characteristics of the nodules; Model 3 included additional covariates that may influence FNA of thyroid nodules, such as position, hand used for FNA, depth, anterior distance, and posterior distance of the nodules. To investigate potential cutoff values for the MD of nodules for FNA, we performed a restricted cubic spline (RCS) analysis with three knots at the 10th, 50th, and 90th percentiles (25). Subsequently, MD was divided into two categories for further subgroup analysis. Statistical significance was set at p < 0.05. All statistical analyses were conducted using R software (version 4.3.1; R Statistical Software, R Foundation for Statistical Computing, Vienna, Austria).

## 3 Results

### 3.1 Baseline information

In present study, 280 TR5 thyroid nodules from 226 patients underwent FNA. Of these, 143 nodules (41.1%, 143/280) were Bethesda VI in cytopathology, and 20 nodules (7.1%, 20/280) were Bethesda V with BRAF V600E mutation, these 163 nodules were categorized as PM group (58.2%, 163/280). The remaining 117 nodules were categorized as PB group (41.8%, 117/280), including 11 nodules with Bethesda I (3.9%, 11/280), 53 with Bethesda II (18.9%, 53/280), 25 with Bethesda III (8.9%, 25/280), 6 with Bethesda IV (2.1%, 6/280), and 22 with Bethesda V without BRAF V600E mutation (7.9%, 22/280).

Demographic, clinical, and imaging data for both groups are detailed in **Table 2**. The PM group exhibited a significantly larger MD of nodules compared with the PB group [Malignant group vs. benign cytopathology group, 6.5mm (5.0, 8.4) > 5.3mm (4.0, 7.0), p < 0.001]. Additionally, the PM group was significantly younger than PB group [Median age: 40 years (range 33-49) vs. 49 years (range 38-58), p < 0.001]. No statistical differences were observed in other variables between the two groups.

**Table 2:** Demographic, clinical and imaging information of probably malignant and probably benign groups.

Variables	Total (n = 280)	Probably Malignant (n = 163)	Probably Benign (n = 117)	p-value
Sex, n (%)				0.205
Female	198 (71)	110 (67)	88 (75)	
Male	82 (29)	53 (33)	29 (25)	
Age (year), Median (Q1,Q3)	42 (34, 54)	40 (33, 49)	49 (38, 58)	< 0.001
Number of FNA, Median (Q1,Q3)	2 (1, 2)	1 (1, 2)	2 (1, 2)	0.961
Composition, n (%)				1
Mixed	1 (0)	1 (1)	0 (0)	
Solid	279 (100)	162 (99)	117 (100)	
Echogenecity, n (%)				0.573
Hypoechoic and very hypoechoic	277 (99)	162 (99)	115 (98)	
Isoechoic	3 (1)	1 (1)	2 (2)	
Shape, n (%)				1
Wider than Tall	67 (24)	39 (24)	28 (24)	
Taller than Wide	213 (76)	124 (76)	89 (76)	
Margin, n (%)				0.153
Smooth	107 (38)	58 (36)	49 (42)	
Ill-defined	91 (32)	50 (31)	41 (35)	
Irregular or extension	82 (29)	55 (34)	27 (23)	
Echogenic foci, n (%)				0.822
None	137 (49)	81 (50)	56 (48)	
Peripheral or Macrocalcification	23 (8)	12 (7)	11 (9)	
Punctate echogenic foci	120 (43)	70 (43)	50 (43)	
Hashimoto thyroiditis, n (%)				1
Yes	56 (20)	33 (20)	23 (20)	
No	224 (80)	130 (80)	94 (80)	
MD (mm), Median (Q1,Q3)	5.9 (4.57, 8.1)	6.5 (5, 8.4)	5.3 (4, 7)	< 0.001
Depth (mm), Median (Q1,Q3)	10.8 (8, 13.62)	10.6 (7.3, 13.5)	11.1 (8.8, 14)	0.075
Anterior distance (mm), Median (Q1,Q3)	2.15 (0, 4.2)	2.1 (0, 3.9)	2.5 (0, 4.5)	0.209
Posterior distance (mm), Median (Q1,Q3)	3.4 (0, 5.93)	3.3 (0, 5.95)	3.6 (0, 5.9)	0.585
Position of nodule, n (%)				0.47
Right lobe	143 (51)	79 (48)	64 (55)	
Isthmus	30 (11)	20 (12)	10 (9)	
Left lobe	107 (38)	64 (39)	43 (37)	
Dominance of the hand used for FNA, n (%)				0.675
Non dominant hand	53 (19)	29 (18)	24 (21)	
Dominant hand	227 (81)	134 (82)	93 (79)	

Malignant Cytopathology Group: Thyroid nodules had Bethesda VI or Bethesda V with Braf V600E mutation in cytopathology.

Probably Malignant Group: Thyroid nodules had Bethesda VI, or Bethesda V with Braf V600E mutation in cytopathology.

Probably Benign Group: Thyroid nodules had Bethesda I, II, III, IV, or Bethesda V without Braf V600E mutation in cytopathology.

FNA: fine needle aspiration.

MD: maximum diameter of targeted nodules

Number of FNA: Number of nodules underwent FNA simultaneously in a patient.

Mann-Whitney U test: age, number of nodules underwent FNA simultaneously, maximum diameter, depth, anterior distance, posterior distance, medial distance, lateral distance.

Chi-square test: sex, composition, echogenicity, shape, margin, echogenic foci, Hashimoto thyroiditis, position, hand used for FNA, Hashimoto thyroiditis.

Bold means significant difference (P<0.05).

### 3.2 Logistics regression

Three binary logistics regression models were constructed to investigated the relationship between MD of thyroid nodules and PM outcomes(**Table 3**). The MD was included in model as a continuous variables or a discontinuous variables [Quartiles 1 (Q1): 2.1-4.6mm; Q2: 4.6-5.9mm; Q3: 5.9-8.1mm; Q4:8.1-40.8mm] respectively.

**Table 3:** Logistics regression model for association between MD and malignant cytopathologY outcomes in FNA of thyroid nodules.

	Model 1		Model 2		Model 3	
	OR (95%CI)	p	OR (95%CI)	p	OR (95%CI)	p
Q1	reference	**–**	reference	**–**	reference	**–**
Q2	3.39 (1.71-6.90)	**0.001**	4.01 (1.95-8.63)	**<0.001**	4.09 (1.93-8.95)	**<0.001**
Q3	2.80 (1.45-5.53)	**0.003**	3.65 (1.74-7.91)	**0.001**	3.93 (1.82-8.77)	**0.001**
Q4	3.54 (1.80-7.11)	**<0.001**	3.99 (1.80-9.15)	**0.001**	4.71 (1.97-11.69)	**0.001**
MD	1.07 (1.02-1.16)	**0.023**	1.08 (1.01-1.18)	**0.042**	1.09 (1.01-1.20)	**0.042**

OR, odds ratio; CI, confidence interval; FNA: fine needle aspiration; MD: maximum diameter of TR5 thyroid nodules.

Quartile of MD: Q1: 2.1-4.6mm; Q2: 4.6-5.9mm; Q3: 5.9-8.1mm; Q4: 8.1-40.8mm.

Malignant cytopathology outcomes: Bethesda VI or Bethesda V with Braf V600E mutation in cytopathology of thyroid nodules.

Bold means significant difference (P<0.05).

Model 1: adjusted for none.

Model 2: adjusted for age, gender and the characteristics of nodules in ultrasound imaging, including echogenicity, shape, margin, echogenic foci and Hashimoto thyroiditis. The echogenecity and composition of nodules was excluded because that almost all thyroid nodules in present study was solid or almost completely solid (100%, 282/283) and hypoechoic/very hypoechoic (99%, 280/283).

Model 3: adjusted for the position of nodule, hand used for FNA, depth, anterior distance, and posterior distance of nodules.

**Figure 4:**
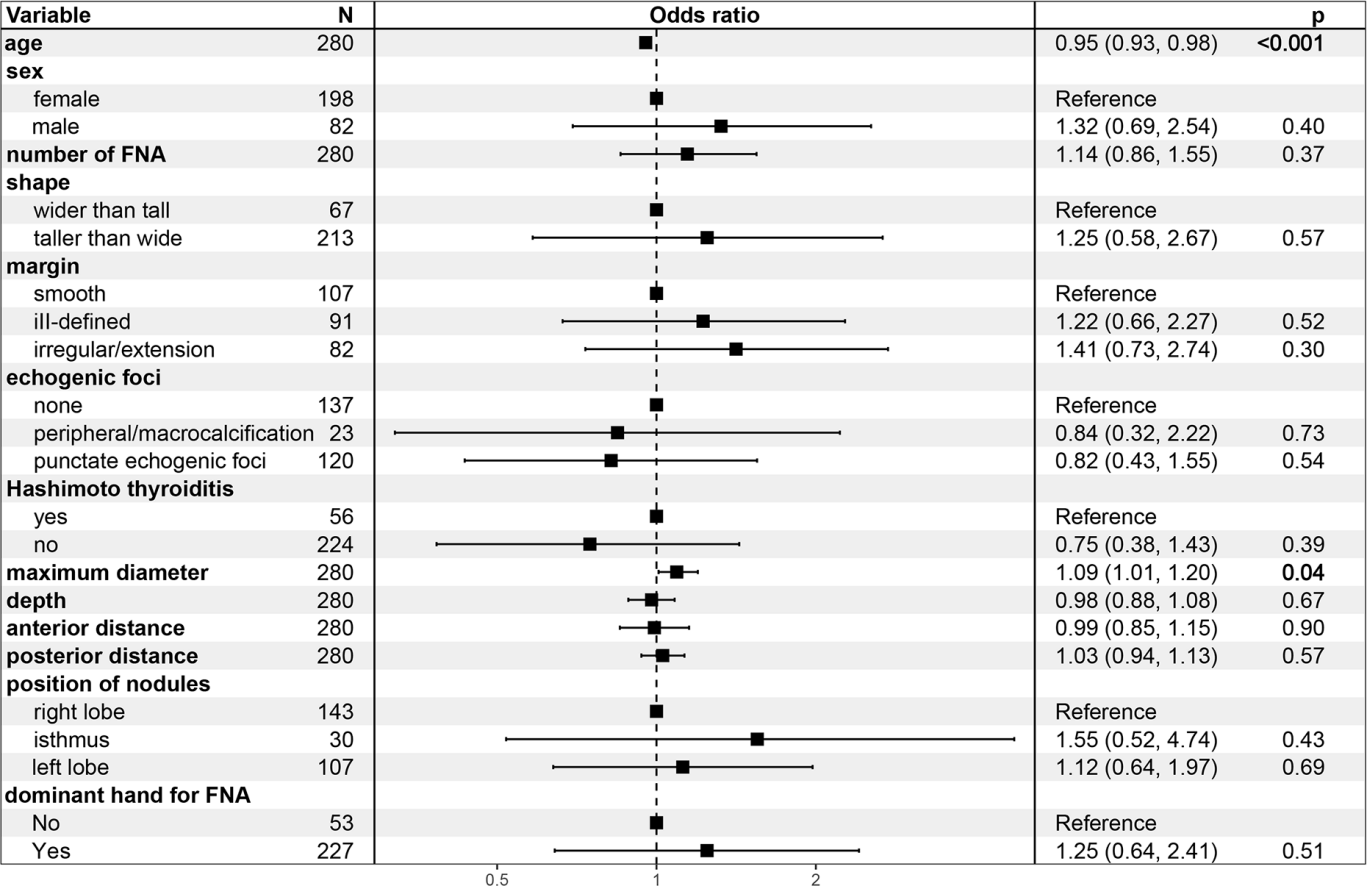
Forest plot for multivariate logistic regression model 3.

In Model 1, the odds ratio (OR) and 95% confidence interval (CI) of MD were 1.07 (1.02-1.16), p=0.023), suggesting an increased likelihood of PM outcomes in FNA with every unit increase in the MD of TR5 thyroid nodules. These relationship persisted in Model 2 [OR (95%CI): 1.08 (1.01-1.18), p=0.042] and Model 3 [OR (95%CI): 1.16 (1.05-1.31), p=0.007] after adjusted for potential influencing factors. The results of Model 3 indicated that age was a negative predictor of PM outcomes in FNA [OR (95%CI): 1.09 (1.01-1.20), p=0.042] (**Figure 4**). Furthermore, when MD was categorized into quartiles, this relationship remained significant. The Q2, Q3, and Q4 quartiles of MD showed a significantly higher probability of PM outcomes compared to the Q1 quartile in FNA of TR5 thyroid nodules (**Table 3**).

### 3.3 Restricted cubic spline

**Figure 5:**
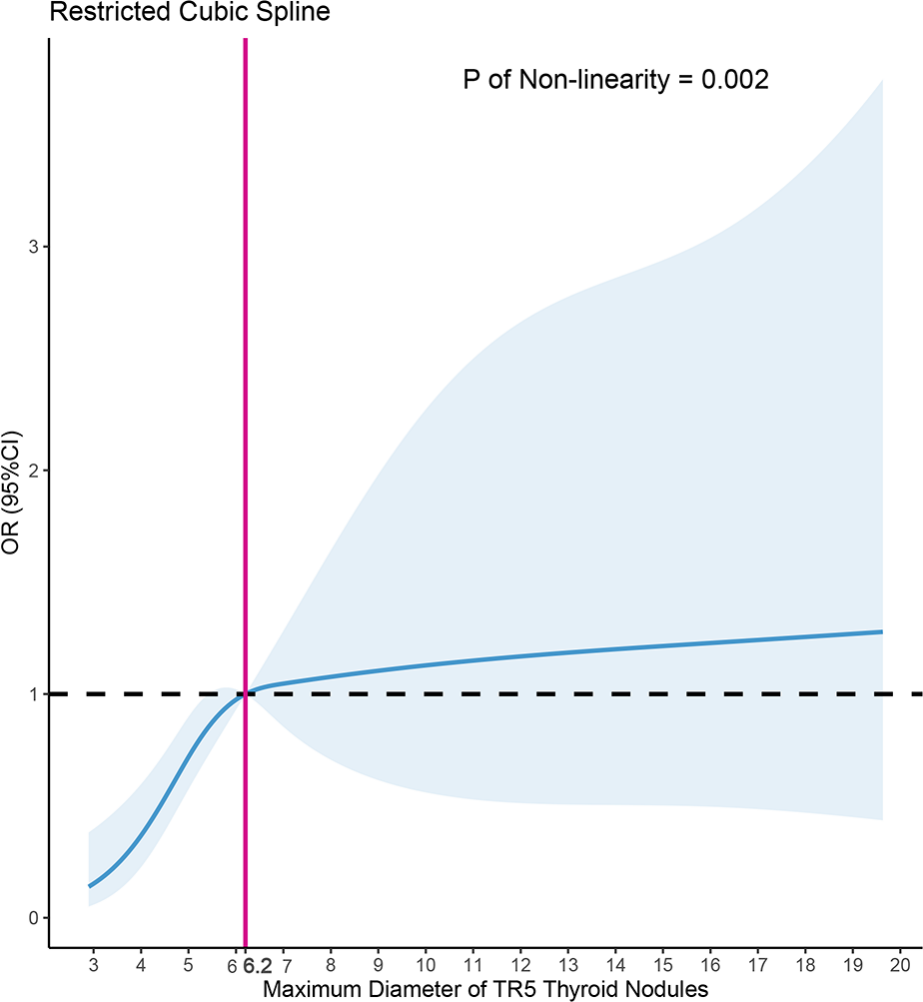
Nonlinear relationship of maximum diameter and malignant cytopathology outcomes in fine needle aspiration (FNA) of ACR TIRADS 5 thyroid nodules, the model was adjusted for age, gender, the shape, margin, echogenic foci of nodules, Hashimoto thyroiditis, position of nodules, dominance of the hand for FNA, depth, anterior distance, and posterior distance of nodules.

Using Model 3 as a basis, we constructed a RCS analysis to delve deeper into the relationship between MD and PM outcomes in FNA (**Figure 5**). A significant non-linear correlation was found between MD and PM outcomes (p=0.002). The inverted L-shaped RCS curve revealed an increasing probability of PM outcomes in TR5 thyroid nodules with higher MD. The RCS analysis for FNA in TR5 thyroid nodules identified 6.2mm as a potential MD cutoff value.

### 3.4 Subgroup logistics regression

Furthermore, TR5 thyroid nodules were categorized into two subgroups based on the results of RCS and the MD of the nodules: those with MD ≥ 6.2mm and those with MD < 6.2mm (**Table 4**). Logistic regression analyses were conducted separately for each subgroup. It was found that the MD of TR5 thyroid nodules was significantly positively correlated with PM outcomes in FNA for nodules with MD < 6.2mm [Odds Ratio (OR) 95% Confidence Interval (CI): 2.43 (1.60-3.86), p < 0.001]. However, this correlation was not significant in nodules with MD ≥ 6.2mm [OR (95% CI): 1.03 (0.94-1.15), p = 0.519].

**Table 4:** Subgroup logistics regression based on restricted cubic spline and maximum diameter.

Subgroup	OR (95%CI)	p
MD ≥ 6.2mm	1.03 (0.94-1.15)	0.519
MD < 6.2mm	2.43 (1.60-3.86)	**<0.001**

OR, odds ratio; CI, confidence interval; MD: maximum diameter of TR5 thyroid nodules.

Bold means significant difference (P<0.05).

## 4 Discussion

FNA is a widely recommended diagnostic tool for determining the nature of the thyroid nodules in most guidelines (2, 6, 9). However, the application of FNA for thyroid nodules smaller than 1cm is debated, with no established MD threshold for suspicious nodules identified (9). Additionally, due to the potential of lymph node metastasis in PTMC and the swift advancement of thermal ablation treatments for PTMC (19–21, 26, 27), FNA for thyroid nodules smaller than 1cm has become increasingly necessary in recent years. To address these issues, this study investigated the relationship between MD and malignant cytopathology outcomes in FNA cytopathology of thyroid nodules, and explored the appropriate MD threshold for TR5 thyroid nodules requiring FNA.

In present study, we summarized 280 TR5 nodules that underwent FNA, with 58.2% (163/280) exhibiting PM outcomes. The MD in the malignant group was 6.5mm (5.0, 8.4), notably larger than the 5.3mm (4.0, 7.0) in the PB group (p < 0.001). The multivariate logistic regression model revealed a positive correlation between MD and PM outcomes [OR (95%CI): 1.09 (1.01-1.20), p=0.042]. Further RCS analysis indicated that an MD of ≥ 6.2mm is a more suitable criterion for TR5 thyroid nodules to undergo FNA.

Remarkably, prior research has not specifically focused on the relationship between the MD of thyroid nodules and malignant outcomes in FNA. Although several studies have explored the relationship between the MD of thyroid nodules and FNA results, they predominantly concentrated on non-diagnostic rather than malignant cytopathology outcomes. Choi et al. suggested that the MD ≤ 5mm positively influenced non-diagnostic results in repeat FNA (OR = 1.742, p = 0.0331) (16). Similarly, studies by Leenhardt et al., Leung et al., Li et al., and Chen et al. observed a comparable relationship in FNA for thyroid nodules (10, 18, 28, 29). Conversely, other studies found no significant correlation between nodule MD and sample adequacy in FNA (12, 15, 30, 31). We believe that nodule MD does not influence sample size in FNA, as operators can obtain thyroid cells surrounding even very small nodules. Consequently, thyroid nodule size does not lead to inadequate sample sizes in FNA, but theoretically could increase the false negative rate. One study found that the sensitivity, specificity, accuracy, and predictive values for nodules ≤ 5mm were lower than those for nodules 6-10mm and >10mm, although the differences were not significant (31). However, another study found that thyroid nodules < 5mm had a significantly higher false negative rate (3.9%) compared to 5 - 10mm (0.9%) and > 10mm (0%) (17).

Furthermore, this study is the first to statistically analyze and identify a suitable MD for thyroid nodules undergoing FNA. We conducted RCS analysis to determine the potential MD threshold for TR5 thyroid nodules undergoing FNA. The inverted L-shaped RCS curve indicated that 6.2mm might be an appropriate MD threshold, a finding further corroborated by subgroup logistic regression analysis. These results suggest an increased likelihood of benign cytopathology outcomes in FNA of TR5 thyroid nodules smaller than 6.2mm, compared to those ≥ 6.2mm. Based on these findings, we propose that an MD ≥ 6.2mm could be a more indicative threshold for FNA. Additionally, for TR5 nodules smaller than 6.2mm with benign cytopathology FNA outcomes, patients should be informed about the elevated risk of false negatives and the need for more aggressive follow-up or treatment, particularly for nodules in high-risk locations, such as the paratracheal position.

Besides MD, numerous studies have identified various factors potentially influencing FNA outcomes in thyroid nodules (10, 12–16, 18, 28–30, 32, 33), including age (14), needle path (18), needle size (11), calcification (10, 12, 18), echogenicity (14, 16), composition (13), shape (12), the depth of nodule (29, 30), and Hashimoto thyroiditis (12) et al. However, these studies primarily focused on factors influencing non-diagnostic outcomes (Bethesda I or III), with few examining the potential factors impacting malignant outcomes in FNA of thyroid nodules.

In fact, the presumption prior to FNA in TR5 thyroid nodules is that the targeted nodules are malignant; therefore, malignant cytopathology outcomes from FNA could be a more appropriate outcome variable. In this study, Bethesda VI and Bethesda V with the BRAF V600E mutation were considered outcome variables, and logistic regression models were constructed. The MD of nodules was identified as the most significant factor influencing malignant cytopathology outcomes of FNA in TR5 thyroid nodules, showing a notably positive correlation in Models 1, 2, and 3. Other potential influencing factors, such as sex, shape, margin, echogenic foci, nodule depth, anterior distance, posterior distance, Hashimoto’s thyroiditis, and the hand used for FNA, were not statistically significant. Interestingly, age demonstrated a negative correlation with malignant cytopathology outcomes in FNA for TR5 thyroid nodules in Models 2 and 3. Similar findings were observed in previous studies (34, 35).

This study had several limitations, the first being its retrospective design, which inherently carries the risk of bias. Second, some potential influencing factors, like the blood supply of thyroid nodules, were not incorporated into our model due to the absence of relevant data, but could be considered in future, well-designed prospective studies. Third, the relatively small sample size and non-normal distribution resulted in wide confidence intervals in the latter part of the RCS analysis.

In conclusion, the MD of TR5 thyroid nodules significantly influences the likelihood of malignant outcomes in FNA, with 6.2mm emerging as a potential MD threshold. The TR5 thyroid nodules with MD of ≥ 6.2mm are thus more appropriately indicated for FNA. Significantly, this study is the first to investigate the potential MD threshold for TR5 thyroid nodules undergoing FNA based on the RCS analysis.

## Data availability statement

The raw data supporting the conclusions of this article will be made available by the authors, without undue reservation.

## Ethics statement

The studies involving humans were approved by Clinical Research Ethics Committee of China-Japan Friendship Hospital. The studies were conducted in accordance with the local legislation and institutional requirements. The ethics committee/institutional review board waived the requirement of written informed consent for participation from the participants or the participants’ legal guardians/next of kin because the examination results and radiological data of patients were published anonymously.

## Author contributions

S-LC: Writing – original draft. W-YS: Methodology, Writing – review & editing. Y-RN: Validation, Writing – review & editing. Z-LZ: Resources, Writing – review & editing. YW: Supervision, Writing – review & editing. JW: Resources, Writing – review & editing. L-LP: Data curation, Writing – review & editing. YL: Data curation, Writing – review & editing. M-AY: Conceptualization, Supervision, Writing – review & editing.
